# A New Predictive Index for Osteoporosis in Men under 70 Years of Age: An Index to Identify Male Candidates for Osteoporosis Screening by Bone Mineral Density

**DOI:** 10.1155/2014/781897

**Published:** 2014-03-03

**Authors:** Lee Oh Kim, Hyeon-Ju Kim, Mi Hee Kong

**Affiliations:** ^1^Department of Family Medicine, Jeju National University Hospital, Aran 13gil 15, Jeju-si, Jeju 690-767, Republic of Korea; ^2^Department of Family Medicine, School of Medicine, Jeju National University, Jeju, Republic of Korea

## Abstract

*Background*. Bone mineral density (BMD) screening guidelines for osteoporosis in men seem to have remained unclear. We aimed to set up a predictive index for the osteoporosis(PIO) in men under 70 years of age and present the optimal cutoff value of it, so that clinicians might use it to identify male candidates who benefit from taking the BMD screening. *Methods*. Adult men under 70 years old who met certain criteria were included. With the determined significant predictors for osteoporosis, we created a new index that presumably best predicts the osteoporosis and compared the predictability of it to other variables. Lastly, the optimal cutoff value of the PIO was calculated. *Results*. A total of 359 men were included. Age, weight, and current smoking status turned out to be significant predictors for osteoporosis. The PIO was as follows: [age(years) + 10 (for current smoker)]/weight(kg). Compared to other variables, the PIO showed the greatest predictive performance with the optimal cutoff point being 0.87 at which sensitivity and specificity were 71.9% and 70.0%, respectively. *Conclusion*. A new predictive index appeared to predict the presence of osteoporosis fairly well and thus can be used with its cutoff point to identify men under 70 years of age who need BMD screening.

## 1. Introduction

Male osteoporosis has been increasingly recognized as a major global health problem over the last decade. Demographically, its incidence is increasing and expected to keep rising given prolonged life span. Specifically, the prevalence of osteoporosis in Korean men aged 50 years or older was 7.5% according to national health survey data [1]. This figure was higher than that of Caucasians and the increasing trends for osteoporosis seemed to have been on steady rise since early twenty-first century. The clinical importance of osteoporosis comes from the fact that fractures associated with osteoporosis show high morbidity and mortality in men compared with women [2]. In this regard, early detection of men with osteoporosis is determined by bone mineral density (BMD), so that they may benefit from early treatment that seems important strategy. Currently, BMD is still considered a single important predictor of fracture risk [3–5] despite a wide use of FRAX, a powerful tool to predict 10-year fracture probability that also includes BMD as one of data to calculate the risk [6]. In this sense, there is no doubt that BMD measurement has significant screening value and now the point is how to identify men who are likely to have osteoporosis and should, therefore, undergo BMD screening. While there appeared to be generally agreed consensus recommendations for osteoporosis screening by BMD in women [7–10], it seems like those guidelines in men vary according to organizations. Some but not all organizations advocate screening men at or older than 70 years of age by DXA and men younger than 70 with risk factors for fracture [11–13]. A few clinical assessment tools have been developed to help clinician to determine the risk of osteoporosis in men and recommend DXA accordingly. Of those, the osteoporosis self-assessment screening tool (OST) has been considered a simple and effective clinical tool to identify men at increased risk of osteoporosis [14–16]. This index has been validated in many Caucasian populations including Koreans [17] as an effective tool to use in determining which men should get BMD measurement. Interestingly, most studies that assessed the validity of OST in men included old individuals, thus excluding young people and setting no upper age limit of the subjects [17–20]. Given that men older than 70 years of age regardless of having risk factors for osteoporosis need to be screened by DXA according to certain guidelines [11–13], maybe we also have to assess the validity of the OST for men below 70 years old. Besides the OST, we have only a few clinical assessment tools that appeared not to be the best options to use in finding men at high risk for osteoporosis. In this regard, we aimed to develop a new clinical, predictive index for osteoporosis and ultimately use it in clinical practice to select Korean men under 70 years of age who should take the BMD screening for osteoporosis. To say that again, this new index was designed to serve as ab easy-to-use tool for clinicians in determining male candidates for osteoporosis screening by BMD test.

## 2. Materials and Methods

### 2.1. Study of Subjects and Anthropometric Measurements

We enrolled subjects who had attended a health promotion center in a Jeju university hospital for diverse health examinations from July 2009 to January 2012. They were generally healthy and interested enough in their health to pay for the exams. The variables taken into consideration at the initial stage of designing of the study were as follows based on a review article by Robert [21]: age; low body weight; low trauma fracture; oral glucocorticoid therapy; androgen deprivation therapy for prostate cancer; the other hypogonadism; smoking and/or chronic obstructive pulmonary disease; excess alcohol intake (>3 units daily); malabsorption, celiac disease; surgery for peptic ulcer disease; bariatric surgery; enzyme-inducing anti-seizure medications; hyperthyroidism; osteopenia/fracture noted on X-ray; and mobility disorders (Parkinson's disease, stroke, multiple sclerosis, etc.). Of those listed, subjects with following uncommon medical conditions based on medical history questionnaire were excluded from the present study: history or evidence of low or atraumatic fractures and history of diseases or taking medications that may affect bone density such as hyperthyroidism, bone diseases, chronic renal failure, rheumatoid arthritis, glucocorticoid use, and bisphosphonate. By the way, the use of calcium and any form of vitamins was not excluded. Finally, those with following risk factors that are common and proven to be significant according to previous studies were included: age, weight, current smoking, heavy alcohol consumption, and exercise. Regarding age, we believed that individuals aged 70 years or older should be candidates for BMD screening regardless of having additional risk factors, and therefore they do not need any risk assessment indices to decide whether or not to undergo BMD measurement. For this reason, subjects aged 70 years or above were excluded from this study. Weights were measured while subjects wore light clothing and no shoes using calibrated electronic scales. Height was obtained by a fixed stadiometer and body mass index (BMI) was calculated as weight in kilograms divided by the height squared in meters. This study was approved by the IRB of Jeju University Hospital (IRB File number 2012-09-003).

### 2.2. Dual Energy X-Ray Absorptiometry (DXA)

The BMD was measured at the lumbar spine (L1~L4) and hip (femoral neck, trochanter, ward) and defined as grams per square centimeter. The BMD measurement was made using a densitometry, GE lunar DPX Bravo (USA). T-score was automatically calculated based on manufacturer's reference data and classified as normal, osteopenia, or osteoporosis according to WHO BMD criteria.

### 2.3. Clinical Assessment Index

The OST was calculated as follows according to the formula of the Osteoporosis Self-Assessment Tool for Asians (OSTA) [22]: [(weight in kilograms − age in years) × 0.2] truncated to an integer.

### 2.4. Statistical Analysis

All statistical analyses were performed using SPSS for window version 17.0 (SPSS Inc., Chicago, IL, USA). Continuous variables were expressed as means and standard deviation using a Student's *t*-test for two groups classified according to the presence or absence of osteoporosis. For categorical data, we used a chi-square to assess differences in means between groups. Logistic regression was performed to identify significant variables in predicting the presence of the osteoporosis. Then using those proven predictors by logistic regression, we developed a clinical assessment model/index through trial and error that best predicted osteoporosis. Receiver operating characteristics (ROC) curve was used to assess predictive accuracy of the variables, the OST, and new predictive index in identifying the osteoporosis in subjects. In order to determine the optimal cutoff value, the point closest to (0,1) on the ROC curve was calculated using the following equation: the minimum value of square root of [(1 − sensitivity)^2^ + (1 − specificity)^2^] [23]. All numbers were truncated to the nearest tenth.

## 3. Results

### 3.1. Characteristics of Subjects

Descriptive characteristics were summarized in Table 1. Our study included 359 men aged 31 to 69 years with a mean age of 54.3 ± 7.9. The mean weight of subjects was 72.2 ± 9.6 and that of T-score was −0.0 ± 1.0. Of the participations, current smokers accounted for 38.7 percent. The prevalence of osteoporosis in our study group was 8.9 percent.  Table 2 summarized the characteristics of two groups stratified according to whether or not they have osteoporosis. Bone density status was defined based on WHO BMD criteria: osteoporosis if the lowest T score either of lumbar spine or femur was less than −2.5; osteopenia if the corresponding figure was between −2.5 and −1.0; normal if the corresponding figure was greater than −1.0. The mean age of normal and osteopenia group and osteoporosis group was 54.0 ± 7.9 and 57.2 ± 7.6, respectively, with statistically significant difference. Likewise, there were significant differences between two groups for weight, BMI, current smoking status, and OST. However, for heavy alcohol consumption and exercise status, other commonly known osteoporosis risk factors, two groups did not show meaningful differences.

### 3.2. Model Development: Predictive Index for Osteoporosis (PIO)

Logistic regression analysis revealed age, weight, and current smoker to be significant variables to predict the presence of osteoporosis in study population both before and after controlling for all five variables: age, weight, current smoker, heavy alcohol consumption, and exercise status (Table 3). Contrary to common expectations, heavy alcohol consumption and exercise status did not prove to be significant predictors for osteoporosis in our study population. The next step was to properly combine those three significant variables to produce a good formula that hopefully better predicted the presence of osteoporosis than each factor did. Based on extensively validated formula of the OST which contains two variables, we tried to merge it with the third variable, status of current smoking, to create a new formula. Basically, we worked on this process by trial-and-error way rather than using specific statistical technique or model in an effort to keep the new index from being complicated formula. Specifically, using the basic combination of age and weight such as the form of “age-weight” or “age/weight,” the status of current smoking was incorporated into the new formula in various integers. The validity of the index under development was examined by calculating sensitivity, specificity, and AUC to determine the appropriate one with both good statistical performance and simplified formula. Finally, we came up with a model that contains three significant variables and retains the simplicity, namely, predictive index for osteoporosis (PIO) [age (years) + 10 (for current smoker)]/weight (kg)], where 10 is added to age in years for current smoker and then is divided by weight in kilogram. Weight was put in kilograms to one decimal place and the PIO was truncated to the nearest hundredth.

### 3.3. Comparison of Diagnostic Performance among Weight, BMI, OST, and PIO

**Figure 1:**
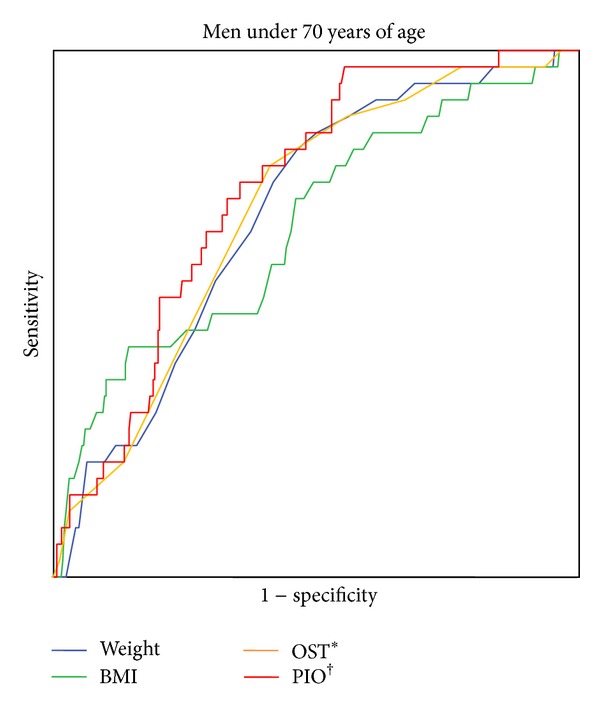
ROC curves of weight, BMI, OST, and PIO to identify osteoporosis in men under 70 years of age. ROC: receiver operating characteristics; BMI: body mass index. *Osteoporosis self-assessment tool [weight (kg) − age (years)] × 0.2, decimal dropped. ^†^Predictive index for osteoporosis [age (years) + 10 (for current smoker)]/weight (kg).

The discriminatory abilities of weight, BMI, OST, and PIO for osteoporosis in men under 70 years of age were evaluated by ROC analysis (Table 4 and Figure 1). All four indices showed significant AUCs in predicting the osteoporosis with the PIO seemingly having the greatest AUC of 0.74 (95% confidence intervals (CI), 0.66–0.81).

However, when it comes to comparing the AUC values of those indices, it turned out that there was no statistical significance of differences between them. On the other hand, according to the traditional academic point system where AUC values lower than 0.7 are considered “poor or worthless,” only PIO appeared to be a fair index for the prediction of osteoporosis in men under 70 years of age [24]. Regarding the optimal cutoff value, that of the PIO was calculated to be 0.87 at which sensitivity and specificity were 71.9% and 70.0%, respectively. In addition, positive and negative predictive values at this optimal cutoff value of 0.87 were 17.2% and 96%, respectively.

## 4. Discussion

Our study developed a new predictive index based on age, weight, and current smoking status to identify Korean men under 70 years of age who are at high risk of having osteoporosis and thus should undergo BMD screening. The PIO appeared to be an easy and simple assessment tool requiring less than 30 seconds to calculate for clinicians to identify men who need BMD screening for osteoporosis. In our study population, a PIO score of 0.87 seemed to serve as the optimal cutoff value which yielded a sensitivity, specificity, positive predictive, and negative predictive values of 71.9%, 70.0%, 17.2%, and 96%, respectively. Given that guidelines about who should be candidates for BMD testing in men vary according to expert groups and appeared not to have reached a consensus, the PIO seems to provide another practical and reliable guide for identifying such candidates. Interestingly, it was noted that the PIO tends to show greater diagnostic performance for osteoporosis in younger adult men. When the utility of the PIO was analyzed according to age groups, the index showed higher discriminatory accuracy for men under 50 years of age (AUC 0.78, 95% CI (0.60–0.96)) than that for men aged 50 to 69 years (AUC 0.70, 95% CI (0.62–0.79)) while the OST did not show any significant performance for men under 50 years of age (AUC 0.65, 95% CI (0.38–0.91)). In other words, the PIO in contrast to the OST appears to show greater diagnostic performance in younger men indicating that such relative superiority of the PIO may decrease in elderly men. Thus, this finding indicates that the PIO may serve as a promising index to identify osteoporosis in young adults although low BMD alone does not necessarily determine the diagnosis of the osteoporosis in young people [25].

The OST has been validated in Asian and Western population as a useful tool in identifying men who need to undergo BMD measurement. Lee et al. assessed the utility of the OST in predicting osteoporosis in Korean men above 50 years of age yielding an AUC of 0.77, sensitivity of 85%, and specificity of 62% [26] for a cutoff value of 0. Likewise, a study by Kung et al. in Chinese men above 50 years using different cutoff (OST < 0) reported an AUC of 0.83 and a sensitivity and specificity of 82% and 67%, respectively [18]. Another study by Li-Yu et al. in Filipinos above men above 40 years of age came up with an AUC of 0.85 and a sensitivity and specificity of 90% and 66%, respectively [20]. Surprisingly, however, our study proved that the OST was ineffective with an AUC of 0.69 in Korean men under 70 years of age. This difference in diagnostic performance of the OST may be attributable to the fact that present study included only individuals aged under 70 years, thus lowering mean age of participants while previous studies have not set upper age limit for participants. Taking into consideration that all men older than 70 years regardless of additional risk factors should be candidates for BMD screening according to some expert groups [11–13], the validity of certain clinical assessment indices need to be assessed for people below 70 years as we did in our study. In this sense, the PIO appears superior to the OST at least for Korean men under 70 years old in terms of their predictability for osteoporosis.

Compared to the OST, better performance of the PIO may be attributable to containing an additional variable, current smoking status, in the formula of PIO. The link between smoking and osteoporosis has long been recognized and a recent study by Tamaki et al. addressed the association of smoking (current or previous) with male osteoporosis [26]. Besides age and weight, the third significant factor, namely current smoking, seems strongly associated with low bone density; thus, adding this variable to a formula theoretically is expected to produce a better predictive index. From this point of view, there is no wonder that chronic obstructive pulmonary disease (COPD) instead of smoking could be considered as another strong predictor of osteoporosis. Actually, a fascinating prediction model called a male osteoporosis risk estimation score (MORES) [27] included a history of COPD as one of three variables (age, weight, and history of COPD) in its formula and showed excellent predictive validity in predicting osteoporosis in American men aged 50 years and older. The MORES yielded an AUC of 0.83 and sensitivity and specificity of 95% and 59%, respectively. Unfortunately, we could not compare the diagnostic performances between the OST, the MORES, and the PIO because of lacking in number of our participants with a diagnosed COPD needed to properly analyze.

Our results must be interpreted within the context of some limitations. Firstly, we did not count all risk factors for osteoporosis in selecting variables to devise a new predictive index. For instance, history of decrease in height or eating habit was not taken into consideration in the modeling process. Ideally, a predictive model should consist of as many independent variables as possible to better predict the outcome. However, it was almost impossible to collect data containing information about all risk factors and also not practical to implement a clinical assessment index consisting of complicated factors. Secondly, this was a single-center study located in an island with a small sample size which limits the generalization of the result to whole population. Nevertheless, we still believe that the new index has a potential for use in the general population. One way to prove its utility is to use it in actual practice and see how well it predicts osteoporosis in men.

In summary, the purpose of this new predictive index was not only to diagnose osteoporosis but also to identify men aged under 70 years who are more likely to have osteoporosis and should, therefore, undergo BMD screening.

To that end, we validated the PIO by comparing the diagnostic performance between the PIO, the OST, weight, and BMI proving that the new index appeared a little bit superior to the OST and other variables in this particular age group. A larger and multi-ethnic population-based study assessing our new predictive index will be needed to validate its utility in population outside Korea.

## Figures and Tables

**Table 1:** Summary of descriptive characteristics of 359 men.

Variables	Mean ± SD or number (%)
Age (years)	54.3 ± 7.9
Weight (kg)	72.2 ± 9.6
Height (cm)	168.8 ± 5.7
BMI (kg/m^2^)	25.3 ± 2.9
Current smoker	139 (38.7)
Heavy alcohol consumption*	206 (57.4)
Exercise^†^	256 (71.3)
*T*-score^‡^	−0.0 ± 1.0
Number of subjects with osteoporosis	32 (8.9)
OST^§^	3.2 ± 2.6

SD: standard deviation; BMI: body mass index.

*Consuming two or more bottles of any kind of alcohol per week.

^†^Exercise of any kind for any duration on a regular basis.

^‡^The lowest *T*-score either of femur or lumbar spine.

^§^Osteoporosis self-assessment tool [weight (kg) − age (years)]  × 0.2, decimal dropped.

**Table 2:** Characteristics of subjects according to bone density status.

	Normal and osteopenia* (*n* = 327)	Osteoporosis* (*n* = 32)	*P* value
Age (years)	54.0 ± 7.9	57.2 ± 7.6	0.028
Weight (kg)	76.7 ± 9.6	67.2 ± 7.5	0.002
Height (cm)	168.9 ± 5.8	168.3 ± 4.2	0.565
BMI (kg/m^2^)	25.5 ± 2.9	23.8 ± 3.0	0.002
Current smoker	120 (36.7)	19 (59.4)	0.012
Heavy alcohol consumption^†^	189 (57.8)	17 (53.1)	0.610
Exercise^‡^	233 (71.3)	23 (71.9)	0.941
OST^§^	3.4 ± 2.6	1.7 ± 2.0	0.001

Data are mean ± standard deviation or number (percent).

*P* values: by independent *t*-test for continuous variables and by chi-square test for categorical variables.

BMI: body mass index.

*Defined according to WHO BMD criteria: with the lowest *T*-score either of lumbar spine or femur equal to or less −2.5 being osteoporosis and a *T*-score between −2.5 and −1.0 being osteopenia.

^†^Consuming two or more bottles of any kind of alcohol per week.

^‡^Exercise of any kind for any duration on a regular basis.

^§^Osteoporosis self-assessment tool [weight (kg) − age (years)]  × 0.2, decimal dropped.

**Table 3:** Results of logistic regression: crude and multivariate-adjusted OR of osteoporosis for risk factors in men under 70 years of age.

	Unadjusted OR (95% CI)	*P*-value	Adjusted OR (95% CI)*	*P*-value
Age	1.06 (1.01–1.11)	0.030	1.05 (1.00–1.11)	0.044
Weight	0.93 (0.90–0.98)	0.002	0.95 (0.91–0.99)	0.019
Current smoker	2.52 (1.20–5.29)	0.014	3.04 (1.35–6.82)	0.007
Heavy alcohol consumption^†^	0.83 (0.40–1.71)	0.610	0.80 (0.36–1.76)	0.570
Exercise^‡^	1.03 (0.46–2.31)	0.941	1.00 (0.43–2.31)	0.999

OR: odds ratio; CI: confidence interval.

*Adjusted for age, weight, current smoker, heavy alcohol consumption, and exercise except where variable itself is being examined.

^†^Consuming two or more bottles of alcohol per week.

^‡^Exercise of any kind for any duration on a regular basis.

**Table 4:** Area under the ROC curve, sensitivity, specificity, and the optimal cut-off value (only of predictive index) of variables to predict osteoporosis in men under 70 years of age.

	AUC (95% CI)	*P* value^‡^	Sensitivity (%)	Specificity (%)	Cut-off value
Weight	0.69 (0.60–0.77)	0.001	75.0	57.8	70.5
BMI	0.66 (0.56–0.77)	0.002	50.0	69.4	24.0
OST*	0.69 (0.61–0.78)	<0.001	78.1	58.4	2.5
PIO^†^	0.74 (0.66–0.81)	<0.001	71.9	70.0	0.87

ROC: receiver operating characteristics; AUC: area under the curve; CI: confidence interval; BMI: body mass index.

*Osteoporosis self-assessment tool [weight (kg) – age (years)]  × 0.2, decimal dropped.

^†^Predictive index for osteoporosis [age (years) + 10 (for current smoker)]/weight (kg)].

^‡^Null hypothesis: true area = 0.5.
